# Langerhans cell histiocytosis with oral manifestations: a rare and unusual case report

**DOI:** 10.4317/jced.50728

**Published:** 2012-10-01

**Authors:** BK. Yashoda-Devi, N. Rakesh, Manjushree Agarwal

**Affiliations:** 1 MDS, Professor and HOD, Department of Oral Medicine and Radiology, M S Ramaiah Dental College and Hospital, Bangaluru; 2 MDS, Associate Professor, Department of Oral Medicine and Radiology, M S Ramaiah Dental College and Hospital, Bangaluru; 3 PG student (MDS), Department of oral medicine and radiology, M.S. Ramaiah Dental College and hospital, Bangaluru

## Abstract

Langerhans cell histiocytosis (LCH), is a rare, proliferative disorder in which the accumulation of pathologic Langerhans cells leads to local tissue infiltration and destruction. We present a case of a 32 years old, completely edentulous female patient who presented with erythema of hard palate, maxillary alveolar mucosa and mucosa over the distobuccal part of mandibular alveolar ridge with foci of ulcerations. Histopathologic features were suggestive of LCH which was confirmed by immunohistochemistry which was CD1a positive, confirmatory for LCH. Bone scan revealed multiple bone involvement. At this stage, disease had already progressed to multisystem involvement with endocrinal abnormalities (primary hypothyroidism and hyperprolactinemia), requiring aggressive treatment. Therefore, this case is a reminder of the possibility of occurrence of this rare disease in the oral cavity which might manifest itself in multiple presentations thus easily leading to the misdiagnosis and therefore, it could be easily overlooked by dentists.

** Key words:**Langerhans cell histiocytosis, immunohistochemistry, bone scan.

## Introduction

Langerhans cell histiocytosis (LCH) is an uncommon disorder of the dendritic Langerhans cell that lacks histologic evidence of malignancy but behaves in an aggressive manner. This disease has had many previous names including eosinophilic granuloma, Hand-Christian-Schuller disease, and Letterer-Siwe disease. A workshop in Philadelphia in 1985 recognized the common histologic and pathologic features of the disease, grouped them under one heading, and the term LCH was adopted (1).

The disease usually occurs during childhood and the incidence is one case per 200000 children per year, but it may also occur later in life (2). Etiology is unknown and various theories suggest a role for environmental, infectious, immunologic, genetic causes, and even some believe that LCH is a neoplastic process (3). Diagnostic criteria have been well defined by the Writing Group of the Histiocyte Society (4). In addition to many therapeutic combinations including surgery, chemotherapy and radiation new therapeutic strategies are represented by monoclonal CD-1a-antibody-therapy and gene transfer into haemopoietic progenitor cells (5).

In this article, we report a case of LCH, which had presented with oral manifestations without any other systemic signs and symptoms. But on complete work up the patient was diagnosed to have LCH with systemic involvement.

## Case Report

A 32 year old female patient, mother of two children, poorly built and nourished reported to our clinics for replacement of her missing teeth. Patient also complained of burning sensation in the mouth since 1 yr. Patient had visited our department 2 years back with the complaint of pain in the gums and mobile teeth. Diagnosis of acute necrotising ulcerative periodontitis was given. Teeth were not in a condition to be saved and all her teeth were extracted on follow up visits. Patient also gave the medical history of amenorrhea after the delivery of her 2nd child 6 years back.

**Figure 1:**
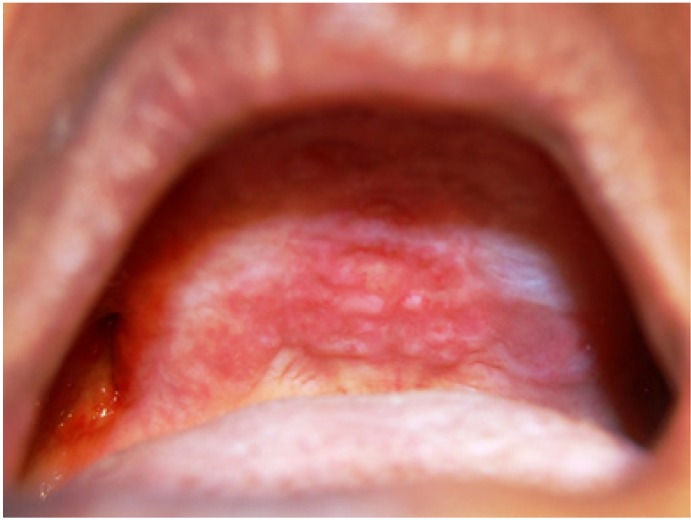
Oral lesions in the hard palate and maxillary alveolar ridge.

Clinical examination showed severely resorbed maxillary and mandibular alveolar ridges. Erythema of hard palate, maxillary alveolar mucosa and distobuccal portion of mandibular alveolar mucosa bilaterally was present with foci of ulcerations (Fig. 1). On palpation mucosa over the hard palate was non tender, soft and oedematous in consistency with no discharge from the lesions. Noma was given as provisional diagnosis and tuberculous ulcer, ulcerative stomatitis secondary to malnutrition, chronic major apthous ulcers and necrotising sialometaplasia were considered as differential diagnosis. Occlusal and panoramic radiograph, routine blood investigations, test to rule out tuberculosis and HIV and incisional biopsy were carried out. Panoramic radiograph revealed extensive bony destruction in the maxilla and mandible. Blood investigations showed increased erythrocyte sedimentation rate and decreased haemoglobin level. White cell count and random blood glucose value were within normal range. Mantoux test was negative and HIV antibody tests were non-reactive.

**Figure 2:**
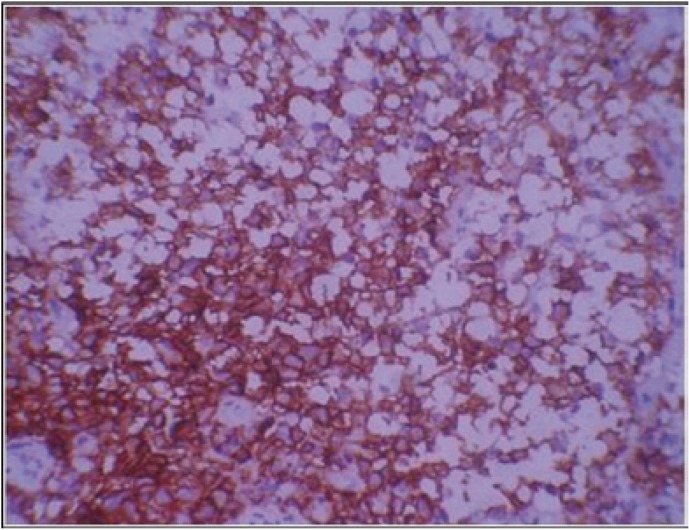
Expression of CD1a antigen in oral mucosal biopsy demonstrating infiltrated Langerhans cells with the cytoplasmic membranous expression of the antigen.

**Figure 3:**
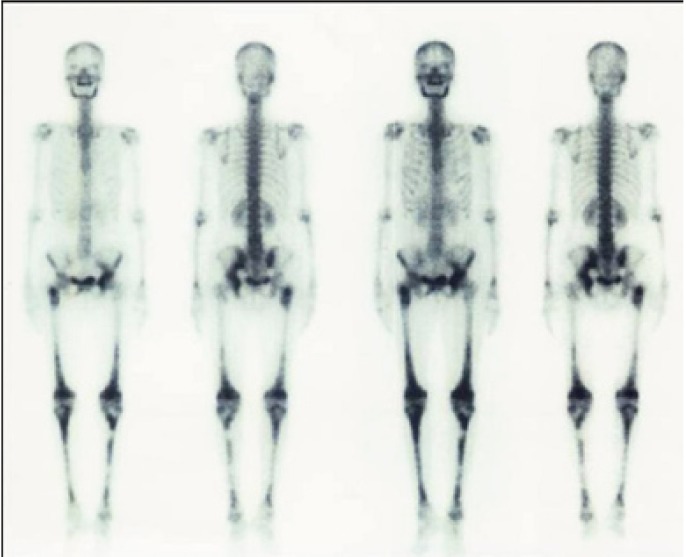
Bone scan was showing increased tracer uptake in the mandible, pelvis: left iliac bone and right pubis, bilateral femori, right proximal femur, bilateral tibiae and abnormal uptake in the lumbar vertebrae.

Histopathologic study revealed diffuse dense chronic inflammatory cell infiltrate and cells with reniform nuclei suggestive of abnormal Langerhans cells in the connective tissue. To confirm the lesional Langerhans cells, immunohistochemical staining for CD1a which is diagnostic for it was performed (6) (Fig. 2). The result was positive and final diagnosis of Langerhans cell histiocytosis was given. To evaluate the generalized bony involvement plain radiography of skull, chest and limbs were done and no abnormality was detected. Bone scan was carried out further and the results revealed increased tracer uptake in the mandible, pelvis: left iliac bone and right pubis, bilateral femori, right proximal femur, bilateral tibiae and abnormal uptake in the lumbar vertebrae (Fig. 3). Endocrine opinion was taken and investigations revealed altered levels of thyroid stimulating hormone, prolactin, follicle stimulating hormone and luteinizing hormone ( Table 1). Patient was diagnosed to have prima-ry hypothyroidism and hyperprolactinemia. Opinion of oncology department was taken and ultrasonography of abdomen was advised. Ultrasonography revealed bulky pancreas with hypoechoic echotexture. Based on histological and other previously mentioned positive investigatory findings the diagnosis of LCH with systemic involvement was established.

**Table 1:**
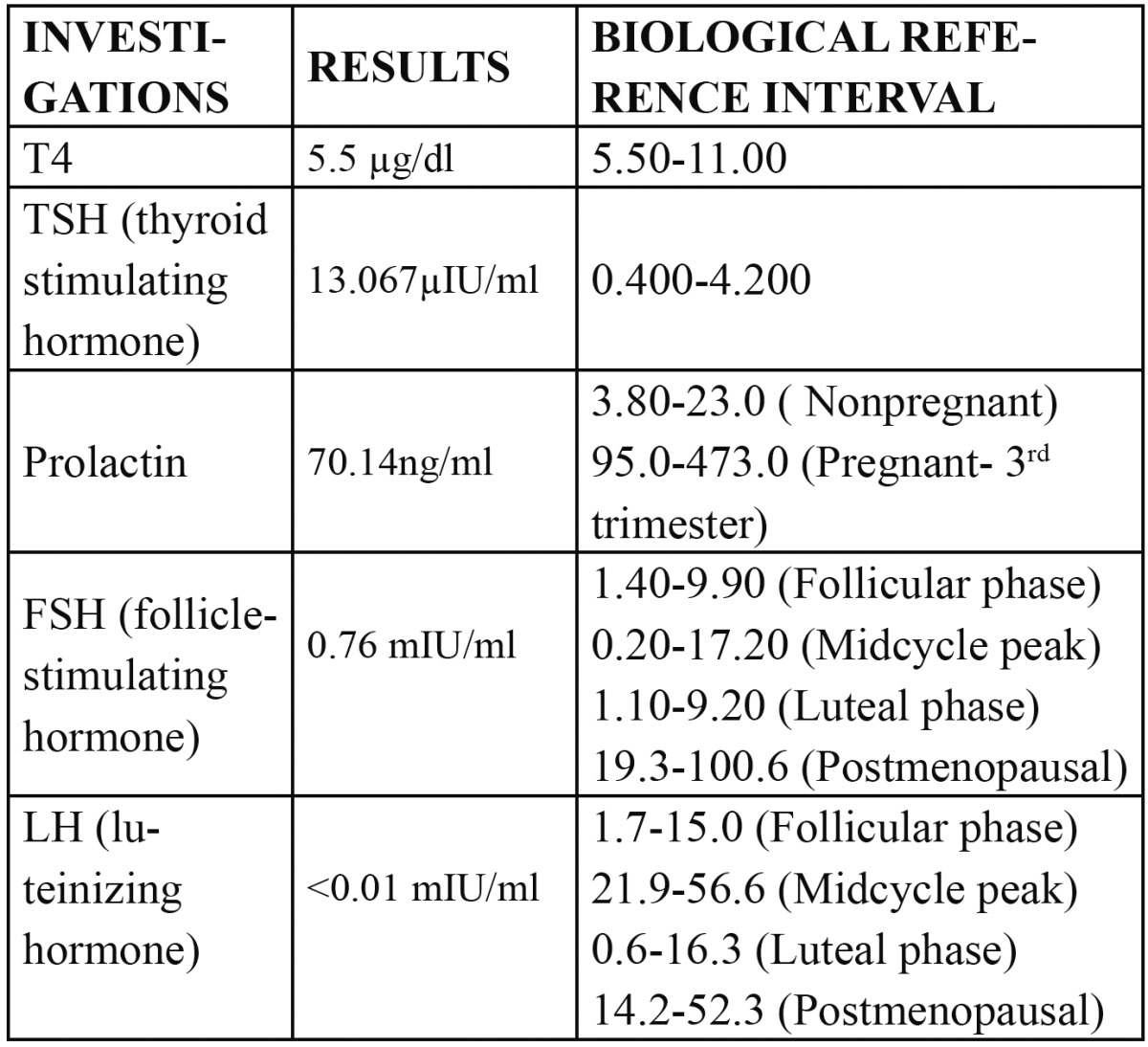
Endocrinological investigations.

Patient was referred to the department of medical oncology. Chemotherapy was planned, including 6 cycles of vinblastine (10 mg weekly) and systemic prednisolone (10 mg thrice a day) for 6 weeks. For oral lesions no treatment was undertaken except for 0.12% chlorhexidine daily rinse. Hormonal replacement therapy was planned after the chemotherapy.

Before third cycle of chemotherapy patient’s total leucocyte count (TLC) fell down to 730/cu mm and granulo-cyte- colony stimulating factor( 300 mcg subcutaneously) was given following which TLC raised to 65,990/cu mm. Third cycle was administered but the patient’s condition deteriorated, patient was brought to the emergency unit in a semiconsciousness state. Her random blood glucose level, blood urea nitrogen, serum creatinine was high and electrolytic imbalance was present. Unfortunately patient succumbed to the complications of the disease.

## Discussion

Langerhans cell histiocytosis is seen rarely in adults, and affects only 1-2/million. Thirty percent of cases present with lesions that affect the jaws. Recognition of this disease is problematic as the clinical and radiographic features are not pathognomonic. Buchmann et al (7) reported that average time to diagnosis was 25 weeks and ranged from 3 to 60 weeks. The oral findings may be pain, mucosal ulceration, gingival necrosis and destruction of alveolar bone with tooth mobility and exfoliation as in our case.

Radiologically, LCH presents as localized, punched-out radiolucencies with no calcification and no sign of scle-rosis or reactions at the borders. There may be severe alveolar bone resorption producing the appearance of teeth ‘‘floating in space’’ (8) as in our case. These non pathognomonic clinical and radiographic features led to the diagnosis of acute nectrotizing ulcerative periodontitis in first place and there was a delay of 90 weeks in diagnosis of LCH.

As LCH lacks pathognomonic clinical or radiographic characteristics, a definitive diagnosis should be based on the histologic and immunohistochemical study of lesional biopsy specimens, where positivity is characteristically observed for CD1a, langerin (CD207) and S-100 protein (9). Because monoclonal proliferation of CD1-positive histiocytes has been shown in all forms of LCH disease, the narrow specificity of the antibody and the ease of the immune staining process render immunostaining for CD1a useful as a routine procedure for diagnostic confirmation of LCH (6). As in our case CD1a immunostaining was positive and hence confirmatory for LCH.

As LCH can be a multifocal disease, radiographic skeletal surveys to be a reasonable procedure for a complete evaluation of the bone condition. In addition, extra skeletal involvement is more likely in patients with multiple bony lesions (10). A bone scintigraphy can also be useful to exclude or to detect additional bone lesions and to follow up patients (9). Full body bone scan in our case revealed multiple bone involvements. Anterior pituitary dysfunction has been described in up to 20% of patients with LCH, usually associated with diabetes insipidus (11). In our case anterior pituitary dysfunction was present without diabetes insipidus.

The treatment of LCH is dependent on lesion size, the degree of tissue involvement and whether it is unifocal or multifocal. Therapy includes surgery, radiation, and chemotherapy, either individually or in combination. Chemotherapy (methotrexate and vinblastine with prednisone) is provided for the more disseminated cases (12). As in our case there was multifocal and systemic involvement, chemotherapy was instituted to the patient. Clinical prognosis of patients become worse with the growing number of involved organs, with rapid disease progression, with limited treatment response and decreasing age of first disease manifestation (8). Recurrence rates depend on the treatment method and location of the lesion and are reported to range from 1.6% to 25% and patients should be closely followed up for a long period of time (10).
